# Analysis of Sentinel Lymph Node Adoption Compared to Systematic Lymphadenectomy in Staging Early Endometrial Cancer at a Tertiary Center: An Ambispective Study

**DOI:** 10.1002/jso.70008

**Published:** 2025-06-23

**Authors:** Rodrigo Pinto Fernandes, Cristina Anton, Marilia Bertolazzi, Maria Luiza Genta, André Lopes, Rossana Veronica Mendonza Lopez, José Antônio Orellana Turri, Rafael Bispo Paschoalini, Ricardo dos Reis, Arnaud Wattiez, Edmund Chada Baracat, Jesus Paula Carvalho

**Affiliations:** ^1^ Setor de Ginecologia Oncológica, Divisão de Ginecologia do Instituto do Câncer do Estado de São Paulo (ICESP) ‐ Hospital das Clínicas Faculdade de Medicina da Universidade de São Paulo (HCFMUSP) São Paulo São Paulo Brazil; ^2^ Divisão de Ginecologia, Instituto do Câncer do Estado de São Paulo (ICESP) ‐ Hospital das Clínicas Faculdade de Medicina da Universidade de São Paulo (HCFMUSP) São Paulo Brazil; ^3^ Departamento de Patologia Instituto do Câncer do Estado de São Paulo (ICESP) São Paulo Brazil; ^4^ Barretos Cancer Hospital Barretos Brazil; ^5^ University of Strasbourg, Strasbourg, France and Latifa Hospital Dubai UAE

**Keywords:** endometrial cancer, indocyanine green, propensity match score, sentinel node biopsy

## Abstract

**Objective:**

The purpose of this study was to assess the impact of changing endometrial carcinoma staging from systematic lymph node dissection to the sentinel lymph node approach.

**Methods:**

This is an ambispective study including patients with endometrial carcinoma (EC) limited to the uterus (FIGO 2018 IA/IB). From December 2015 to October 2021, a group of patients underwent systematic staging with lymph node dissection (LND). From December 2021 to April 2024, another group of patients underwent surgical staging with the sentinel lymph node‐indocyanine green (SLN) algorithm and pathology ultrastaging analisys. The groups were matched (1 LND: 1 SLN) based on age, body mass index (BMI), tumor type, tumor size, and myometrial invasion. The primary endpoints were lymph node involvement, length of surgery, and complications. Complications were classified according to the Common Terminology Criteria for Adverse Events (CTCAE) v5.0.

**Results:**

Two hundred fifty‐seven patients were surgically treated during the study period (156 in the LND cohort, 101 in the SLN cohort). Propensity score matching revealed two equivalent groups containing 84 patients each. The rate of positive lymph nodes was similar between the LND group (3.6%) and the SLN group (8.3%) (OR: 2.46, 95% CI: 0.61–9.84; *p* = 0.205). The length of surgery was significantly lower in the SLN group (152.2 ± 51.9 min) compared to the LND group (304 ± 77.8 min) (*p* < 0.001). Intraoperative blood loss greater than 100 mL was significantly lower in the SLN group (9.5%) compared to the LND group (29.8%) (*p* < 0.001). CTCAE grades requiring intervention (grades 3, 4, and 5) were higher in the LND group (14.3%) compared to the SLN group (4.8%) (*p* = 0.049).

**Conclusion:**

The transition from LND to SLN approach was similar compared to systematic lymphadenectomy, allowing the reduction of surgical length, blood loss and severity of complications without compromising surgical complications and lymph node positivity.

## 1 Introduction

The evaluation of lymph node status, emphasized by FIGO since 1988, not only optimizes adjuvant therapy indications but also potentially eliminates unnecessary treatments when lymph nodes are disease‐free [1, 2]. Despite these benefits, systematic lymphadenectomy (LND) can lead to complications such as lymphocysts, lymphatic ascites, and lymphedema in about 3.4% of cases [3]. While lymph node evaluation is crucial, the number of nodes removed does not significantly impact overall survival, particularly in stage IIIC [4, 5].

The sentinel lymph node (SLN) technique has gained acceptance in early‐stage endometrial cancer (EC). A more accurate and less morbid lymphatic approach when compared to systematic lymphadenectomy [6]. A systematic review demonstrated higher detection rates when transitioning from systematic LND to SLN mapping [7]. The adoption of a SLN algorithm including indocyanine green (ICG) staining and ultrastaging methods has significantly decreased false‐negative rates from 14.9% to 1.9% [8, 9]. Advanced imaging systems have improved detection rates to 95.5% with ICG compared to 61% with other dyes [8]. The learning curve for surgeons is significant, with detection rates improving notably after 20 to 30 cases [10, 11]. Detection failures are often linked to lymphatic channel obstruction, obesity, and the use of blue dye alone [12]. Systematic lymphadenectomy is recommended if SLN detection fails on one side [13]. The absence of lymph node tissue in final pathology is more common during a surgeon's early learning curve but decreases significantly with experience. Ultrastaging has further enhanced lymph node assessment, doubling metastasis detection compared to conventional methods [14, 15]. Comparative studies have confirmed the efficacy of the SLN algorithm over lymph node dissection (LND) in patients with high‐risk EC without suspicious lymph nodes [16]. The SHREC study highlighted the high accuracy of the SLN algorithm in high‐risk tumors, with a sensitivity of 98% and a negative predictive value of 99.5% [17]. Ongoing studies like ALICE aim to further validate these findings, ensuring that the omission of LND does not compromise oncological outcomes [18].

In summary, the SLN technique has become a pivotal component in the surgical staging of EC, providing high detection rates, reducing unnecessary LND‐related complications, and supporting effective adjuvant therapy decisions. Mastery of this technique, along with an understanding of its nuances and challenges, is essential for optimizing patient outcomes in EC treatment. This study aimed to assess the impact of transitioning from systematic lymphadenectomy to the sentinel lymph node (SLN) approach for lymph node staging in endometrial carcinoma.

## 2 Materials and Methods

Between January 2015 and April 2024, patients diagnosed with EC limited to the uterus (FIGO 2018 IA/IB) were surgically treated at our institution. Patients with disease outside the uterus, as identified by CT scan or MRI, were excluded. Two different surgical approaches were analyzed. From January 2015 to October 2021, patients underwent classic staging surgery, which included inspection, hysterectomy, bilateral salpingo‐oophorectomy, and systematic pelvic and paraaortic lymphadenectomy (LND), as previously published [19]. Surgeries were performed using either laparoscopy or robotic approaches (Da Vinci Si, Intuitive Surgical, Sunnyvale, California, US). Specimens were subjected to classic pathological evaluation. From December 2021 to April 2024, patients underwent the SLN algorithm, which included inspection, hysterectomy, bilateral salpingo‐oophorectomy, SLN mapping and ultrastaging pathological evaluation. A total of 4 milliliters of indocyanine green (ICG, Ophthalmos, São Paulo, Brazil) at a concentration of 1.25 mg/mL was injected into the cervix, with 1 milliliter infused superficially and 1 milliliter infused deeply at the 90 and 270‐degree positions at the beginning of the surgical procedure. Dye progression was carefully traced from the paracervix to the first lymph node at the pelvic sidewall and/or presacral and paraaortic areas. The upper paracervical pathway (UPP) towards the pelvic sidewall and/or the lower paracervical pathway (LPP) through the presacral/paraortic area were systematically evaluated. Bilateral or side‐specific reinjection was performed if the ICG failed to spread into the lymphatics after 10 min. Pelvic lymphadenectomy was conducted if reinjection failed on the specific side. A SLN was defined as an ICG‐positive node with an afferent lymphatic channel or as an ICG‐positive lymphatic channel ending in an ICG‐negative node, in the absence of other ICG‐positive nodes along the pathway. If parallel channels were detected, two SLNs were considered. Bulky or suspected lymph nodes were removed separately. Surgeries were performed using either laparoscopy (PINPOINT, Novadaq, Burnaby, British Columbia, Canada) or robotic surgery (Firefly, Da Vinci Si, Intuitive Surgical, Sunnyvale, California, US). Specimens were subjected to the institutional ultrastaging protocol, which included additional three‐level cuts and immunohistochemistry evaluation using CK AE1/AE3. The study was approved by the Institutional Review Board and Ethics Committee (Instituto do Câncer do Estado de São Paulo CCEP 1778/20 – Plataforma Brasil CEP 4.554.839). All participants signed informed consent before surgery. Main data collected included age, BMI, comorbidities, type of tumor, mismatch repair enzymes, myometrial invasion, SLN mapping detection rates and locations, LND and SLN positivity, length of surgery, blood loss and adverse effects accordingly to Common Terminology Criteria for Adverse Events (CTCAE) v5.0 [20].

### 2.1 Statistical Analysis

The calculation of power and sample size was performed using results from studies that evaluated the number of positive lymph nodes detected by the traditional lymphadenectomy technique and the sentinel lymph node technique [7, 21]. Based on the average number of lymph nodes detected by the two techniques across all included studies, for a sample power of 80% and a significance level of *p* = 0.05, the minimum calculated number of patients was 77. Absolute and relative frequencies were calculated for qualitative variables. Measures of central tendency (mean, median) and dispersion (standard deviation, minimum, and maximum values) were calculated for the quantitative variables. Pearson's chi‐square test and Fisher's exact test were used to evaluate the association between qualitative variables. The comparison of quantitative variables between two groups was evaluated using the Student's *t*‐test or the non‐parametric Mann‐Whitney test, as appropriate. Propensity score matching (PSM) was used to diminish potential bias in the comparisons between groups. Variables selected for PSM included age, BMI, histological type, tumor size, and myometrial invasion. To calculate the propensity score, binary logistic regression was used. The probabilities were calculated considering the type of surgery as the outcome (control group: LND and intervention group: SLN). Pairs (1:1) were formed following the nearest neighbor matching method, with a tolerance of 0.01, preferably selecting cases with exact propensity scores. After matching, with similar groups by type of surgery, the association between the type of surgery and lymph node status was evaluated using logistic regression. The significance level adopted was 5%. The analyses were carried out using SPSS for Windows v.25 software.

## 3 Results

All 257 patients included in the study confirmed to have EC limited to the uterus at final pathology. A total of 156 and 101 patients underwent LND and SLN mapping, respectively After propensity score matching two groups of 84 patients were formed (Table 1). With the inclusion of 101 patients in the initial evaluation and 84 patients after propensity score matching, the sample power was 94% and 89%, respectively, with a significance level of *p* = 0.05. In the SLN group, successful bilateral mapping occurred in 89/101(88.1%). Unilateral mapping was achieved in 10/101(9.9%) patients.

**Table 1:** Clinical characteristics according to the type of surgery performed before matching (PSM).

Characteristics	Type of surgery		*p* value
	Systematic lymphadenectomy	Sentinel lymphnode	
	*n* = 156	*n* = 101	
	*n* (%)	*n* (%)	
Age at surgery (years)			0.014^a^
Mean (SD)	62.8 (7.5)	65.3 (8.3)	
Median (min‐max)	62.4 (37.7–82.4)	64.9 (41.9–83.4)	
Body mass index (BMI) (kg/m^b^)^¥^			0.658^b^
Mean (SD)	31.4 (6.1)	32.1 (7.6)	
(min‐max)	30.6 (16.4–53.7)	31 (17.8–52.5)	
Arterial hypertension^¥¥^			0.030^c^
No	73 (47.4)	34 (33.7)	
Yes	81 (52.6)	67 (66.3)	
Diabetes mellitus^¥¥¥^			0.040^c^
No	118 (75.6)	63 (63.6)	
Yes	38 (24.4)	36 (36.4)	
Obesity			0.717^c^
No	70 (44.9)	43 (42.6)	
Yes	86 (55.1)	58 (57.4)	

*Note:* ^¥^One case without information; ^¥¥^two cases without information; ^¥¥¥^31 cases without information.

Abbreviations: min, minimum value; max, maximun value; SD, standard deviation.

^a^ Student's *t*‐test.

^b^ Mann‐Whitney's test.

^c^ Pearson qui‐square.

Failures occurred in 22/101 patients, where reinjection was successful in 12/22 (54.5%) cases. Side specific lymphadenectomy following reinjection failure was performed in 8/101 (7.92%) of cases, two of them bilateral (1.98%). Empty package occurred at 3 (2.98%) cases, unilateral. Our overall and bilateral detection rates for SLN confirmed by pathology was 98.01% and 88.1%, respectively. The average BMI was 31.8 in both groups, both above 30 kg/cm^2^ as previously mentioned. Of the 14 patients who had mapping failure, 35.7% were in the left hemipelvis, 42.85% in the right hemipelvis and 24.4% bilateral. Of the 9 cases of failure on the left, reinjection failed in one case. Of the 10 cases of failure on the right, reinjection failed in 5 cases. Of the three cases of bilateral initial failure (BMIs 34.1; 43.7 and 50 kg/m^2^), reinjection failed in two cases. The only patient in whom the SLN was not identified bilaterally had a BMI of 50.0 kg/m^2^. In total, 108 hemipelvises were bilaterally mapped. Of these, we identified absence of lymph nodes (empty package) in two cases (1.98%).

Final pathology analysis revealed no differences in tumor type between groups (*p* = 0.505). Endometrioid carcinoma was found in 141 cases (69 LND and 72 SLN) followed by 19 serous (12 LND and 7 SLN), and 5 clear cell (3 LND and 4 SLN). Average size of tumor was 4.1 cm in both groups (*p* = 0.520). Myometrial invasion was < 50% in 56 cases in each group and ≥ 50% in 28 cases in each group (*p* = 0.999) (Table 2). Metastasis was present in 3 (3.6%) patients in the LND group and 7 (8.3%) in the SLN group OR 2.46 (0.61–9.84); *p* = 0.205 (Table 3).

**Table 2:** Post surgical and anatomopathological characterístics according to the type of surgery performed after matching (PSM).

Characteristics		Type of surgery		*p* value
		Systematic lymphadenectomy	Sentinel lymphnode	
		*n* = 84	*n* = 84	
		*n* (%)	*n* (%)	
Type of tumor	Endometrioid	69 (82.1)	72 (85.7)	0.505^a^
	Serous	12 (14.3)	7 (8.3)	
	Clear cell	3 (3.6)	4 (4.8)	
	Dediferentiated	0	1 (1.2)	
Size of tumor	Mean (SD)	4.1 (1.9)	4.1 (2.5)	0,520^b^
	Median (min–max)	4 (0–10)	3.7 (0.2–12.1)	
Myometrial invasion	< 50%	56 (66.7)	56 (66.7)	0,999^c^
	≥ 50%	28 (33.3)	28 (33.3)	
Grade	Grade 1	39 (46.4)	40 (47.6)	0,861^c^
	Grade 2	25 (29.8)	22 (26.2)	
	Grade 3	20 (23.8)	22 (26.2)	
Surgical approach	Laparoscopy	63 (75.0)	77 (91.7)	0.004^c^
	Robotics	21 (25.0)	7 (8.3)	
	Open	0	0	
Lymphvascular space invasion (LVSI)	Abscent	80 (95.2)	69 (82.1)	0.007^c^
	Present	4 (4.8)	15 (17.9)	
Cervical stroma invasion	Abscent	82 (97.6)	84 (100)	0.497^a^
	Limited evaluation	2 (2.4)	0	
FIGO staging	IA	43 (51.2)	52 (61.9)	0.182^a^
	IB	26 (31.0)	17 (20.2)	
	II	7 (8.3)	6 (7.1)	
	IIIA	4 (4.8)	2 (2.4)	
	IIIB	1 (1.2)	0	
	IIIC1	1 (1.2)	6 (7.1)	
	IIIC2	2 (2.4)	1 (1.2)	
Length of surgery (min)	Mean (SD)	304.0 (77.8)	152.2 (51.9)	< 0.001^b^
	Median (min–max)	310 (180–560)	147.5 (75–360)	
Blood loss (mL)	0–100	59 (70.2)	76 (90.5)	< 0.001^c^
	101–500	25 (29.8)	8 (9.5)	
p53	Wild	58 (81.7)	70 (83.3)	0.788^c^
	Mutated	13 (18.3)	14 (16.7)	
MMR deficiency	No	50 (65.8)	71 (86.6)	0.002^c^
	Yes	26 (34.2)	11 (13.4)	
MLH1	Abscent	10 (13.0)	6 (7.3)	0.235^c^
	Present	67 (87.0)	76 (92.7)	
MSH2	Abscent	15 (19.5)	4 (4.9)	0.005^c^
	Present	62 (80.5)	77 (95.1)	
MSH6	Abscent	19 (24.7)	3 (3.6)	< 0.001^c^
	Present	58 (75.3)	80 (96.4)	
PMS2	Abscent	13 (16.9)	8 (9.6)	0.175^c^
	Present	64 (83.1)	75 (90.4)	

Abbreviation: NA, not accessible.

^a^ Fisher's exact test.

^b^ Teste de Mann‐Whitney.

^c^ Pearson's chi‐square.

**Table 3:** Lymph node status and type of surgery after propensity match score (PSM).

Characteristics	Lymphnode status		OR (95% CI)	*p* value
	Negative	Positive		
	*n* = 158	*n* = 10		
	*n* (%)	*n* (%)		
Type of surgery				
Systematic lymphdenectomy	81 (96.4)	3 (3.6)	1	
Sentinel lymphnode	77 (91.7)	7 (8.3)	2.46 (0.61–9.84)	0.205
Macrometastasis		2		
Micrometastasis		5		
Isolated tumor cells (ITC)*		5		

Abbreviations: 95% CI, confidence interval of 95%; OR, odds ratio.

* Isolated tumor cells are not considered as metastasis.

Complications according to CTCAE were similar between groups being 18/84 at the LND group and 16/84 at the SLN group (*p* = 0.086). When stratified by severity accordingly to CTCAE (grades 1, 2 vs. grades 3, 4 and 5) severe adverse effects were higher in the LND group: 12 (14.3%) versus 4 (4.8%) (*p* = 0.049). One death (CTCAE grade 5) occurred in each group due to complications of ischemic diverticulum (Table 4). Blood loss higher than 100 mL were significantly lower in the SLN 8 (9.5%) compared to the LND group 25 (29.8%) (*p* < 0.001).

**Table 4:** Surgical complications accordingly to the type of surgery (PSM).

		Type of surgery		*p* value
		Systematic lymphadenectomy	Sentinel lymphnode	
		*n* = 84	*n* = 84	
		*n* (%)	*n* (%)	
CTCAE up to 45 dias				0.086^a^
No complication		66 (78.6)	68 (81.0)	
Grade 1		2 (2.4)	2 (2.4)	
Grade 2		4 (4.8)	10 (11.9)	
Grade 3		6 (7.1)	3 (3.6)	
Grade 4		5 (6.0)	0	
Grade 5		1 (1.2)	1 (1.2)	
Sem complicações		66 (78.6)	68 (81.0)	0.049^b^
Grade 1–2		6 (7.1)	12 (14.3)	
Grade 3–4–5		12 (14.3)	4 (4.8)	
Description of complications				
Intestinal (*n* = 5)	Grade 1	0	1 (25.0)	
	Grade 2	0	2 (50.0)	
	Grade 5	1 (100)	1 (25.0)	
Scar (*n* = 4)	Grade 1	0	1 (50.0)	
	Grade 2	0	1 (50.0)	
	Grade 3	1 (50.0)	0	
	Grade 4	1 (50.0)	0	
Infectious (*n* = 8)	Grade 2	0	5 (83.3)	
	Grade 3	2 (100)	1 (16.7)	
Lymphatic (*n* = 2)	Grade 1	1 (50.0)	0	
	Grade 2	1 (50.0)	0	
Neurologic (*n* = 4)	Grade 2	1 (50.0)	1 (50.0)	
	Grade 3	1 (50.0)	1 (50.0)	
Urologic (*n* = 4)	Grade 2	0	1 (50.0)	
	Grade 3	2 (100)	1 (50.0)	
Vascular (*n* = 7)	Grade 1	1 (14.3)	0	
	Grade 2	2 (28.6)	0	
	Grade 4	4 (57.1)	0	

^a^ Fisher's exact test.

^b^ Chi‐square test.

## 4 Discussion

### 4.1 Summary of Main Results

The present study demonstrated that the transition from systematic surgical staging to a sentinel lymph node‐based approach was successful in our institution. This transition allowed for a reduction in surgical duration, blood loss, and severity of complications without compromising lymph node positivity.

### 4.2 Results in the Context of Published Literature

The role of lymph node assessment, adopted after the 1988 FIGO report, remains the subject of extensive discussion. The complete removal of lymph nodes from the paraaortic and pelvic chains is a highly complex procedure, associated with considerable morbidity and higher complication rate. While lymphadenectomy serves as a valuable tool for adjuvant therapy decisions, its therapeutic role in early‐stage tumors is widely debated. Previous studies evaluating patients with EC restricted to the uterus have demonstrated no significant benefits from performing LND [21, 22]. However, both studies were criticized for their methodology, leaving the therapeutic role of LND in EC unresolved. Sentinel lymph node evaluation, a highly sensitive and accurate method for lymph node assessment, with lower morbidity, appears to be a suitable option for early‐stage EC [21].

**Figure 1:**
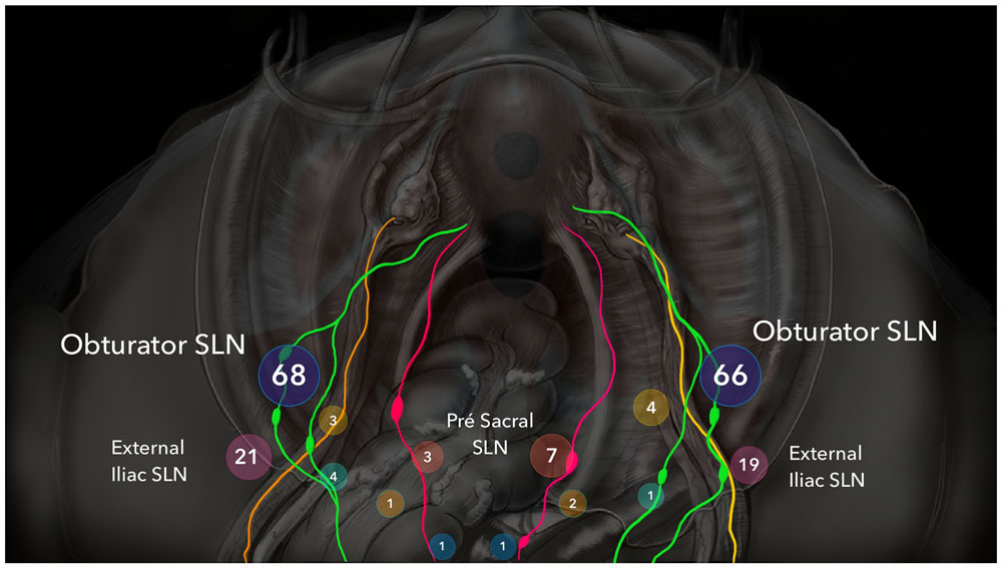
Distribution of sentinel lymph nodes.

We found an overall SLN detection rate of 98.01% and a bilateral detection rate of 88.1%. High detection rates when transitioning from systematic lymphadenectomy to sentinel lymph node exploration are uncommon. Detection rates vary in the literature and tend to increase with the experience of each group [11, 19, 23, 24]. Adherence to SLN protocols and surgical competency assessment tools are strictly necessary to increase detection and diminish failure and empty packages. In our study, the high paracervical lymph node chain was the most frequently mapped, with the obturator lymph node being the most prevalent. Specifically, 65.3% had mapped lymph nodes in the right hemipelvis, and 67.3% had them in the left hemipelvis, averaging 66%. These findings align with those of several authors who also found the obturator lymph node to be the most prevalent, occurring in 52% of cases [24, 25]. Other authors have also reported that the SLN on the inner surface of the external iliac nodes is the most common, found in 55.2% of cases, followed by the obturator lymph node (22.9%) [19]. These locations are close together, as demonstrated in the diagram published by the authors, which may lead to different interpretations of the same location. Lower paracervical pathways are not commonly identified lymphatic routes but were found to be present in 55% of cases, with bilateral LPP present in 33% of cases [23]. We identified lower paracervical pathways in 12.9% of cases on the right and 7.1% on the left, a significantly lower frequency that could be related to the learning curve. In the previously mentioned study, the authors also performed fundal injection, a method no longer advocated by previously published protocols [13]. Even though inconsistent, the presence of lower paracervical pathways must be systematically investigated. Variations in lymphatic channels, interconnections, and the location of SLNs, along with the need for meticulous dissection, have been previously demonstrated [23, 26]. In our series, a patient with FIGO IA endometrioid adenocarcinoma presented with micrometastasis in the left lower paracervical pathway channel and isolated tumor cells in the left upper paracervical pathway channel. This case demonstrates the importance of systematically investigating lower paracervical pathways lymph nodes, which are not included in classic pelvic lymphadenectomy. If these pathways had not been investigated, the micrometastasis would not have been diagnosed, and adjuvant therapy would not have been indicated (Figure 1).

The depth of lymph nodes at the pelvic sidewall is another important factor to be considered [19]. As previously reported and consistent with other studies, the most commonly identified SLN in our study was the obturator lymph node [24, 25]. Success in fluorescence mapping can be influenced by factors such as light incidence, wavelength, penetration depth, and indocyanine green concentration. Two authors reported significant lower rates of detection and SLN mapping in patients with a BMI above 30 and 40 [6, 12]. For every 5‐unit increase in BMI, they found a 1.156‐fold increase in the risk of mapping failure, resulting in a decrease in global and bilateral lymph node detection. The occurrence of “empty packages,” where the surgeon is certain a lymph node was removed but the final anatomical pathological assessment reveals no lymph node tissue, was present in 8.2% of cases [6]. We identified 1.98% of empty packages in our study, a lower percentage compared to Gedgaudaite et al., who reported 10.5% of cases. In our study, patients considered for the propensity score‐matched groups had BMIs ranging from 18.2 to 50.0 kg/m². Although obesity is an important factor that directly influences the detection of lymphatic channels, it is not the only factor. Hyper concentration of indocyanine or confluence of lymphatic channels are some factors that can affect the identification of local lymph nodes. When in doubt, intraoperative frozen section examination is a valuable tool that can indicate the presence of lymphatic tissue and does not tend to harm the definitive anatomical pathological examination and ultrastaging [27].

The morbidity of LND has always been questioned, particularly concerning the extent of the procedure. In addition to the lower limb edema reported by numerous authors, intraoperative complications, especially vascular ones, which increased the risk of death, have also argued against the procedure. In our study, complications were evaluated up to 45 days after surgery, a factor that potentially excludes the development of lower limb edema, which may appear later. When adverse events were measured, no overall difference was found, but when separated by severity (grades 1 and 2 vs. grades 3, 4, and 5), there was statistical significance favoring the SLN group. This finding aligns with Accorsi et al., who found fewer complications and less blood loss when comparing sentinel mapping with systematic lymphadenectomy. In our study one death occurred in each group. In both cases, complication was due to intestinal perforation resulting from the rupture of colonic diverticula.

### 4.3 Strength and Weakness

The main strength of this study was the comparison of both techniques performed by the same team of surgeons, all of whom have high‐volume experience in oncological surgery. Limitations include the retrospective nature of the study and the resulting low number of rare outcomes, such as positive nodes and complications. Additionally, the short follow‐up period may have impaired the potential observation of lymphedema development. Despite the limited experience with SLN mapping, we achieved a high rate of SLN detection. However, this brief experience also allowed for the analysis of an unbiased learning curve within a single institutional surgical team.

### 4.4 Implications for Practice and Future Research

The present study introduced ICG‐based SLN mapping in our institution, promoting a non‐inferior and less morbid method for the surgical treatment of patients with EC at initial stages.

## 5 Conclusion

In this study, we demonstrated that the transition from the LND approach to a SLN algorithm was non inferior when compared to the systematic lymphadenectomy. The SLN approach reduced total surgical length, blood loss, and the severity of complications, without compromising surgical staging.

## Author Contributions

Conception: Rodrigo Pinto Fernandes, Cristina Anton, Jesus Paula Carvalho. Design: Rodrigo Pinto Fernandes and Jesus Paula Carvalho. Development: Rodrigo Pinto Fernandes, Jesus Paula Carvalho, Cristina Anton. Data analysis: Rodrigo Pinto Fernandes, Rossana Veronica Mendonza Lopez, José Antônio Orellana Turri, Cristina Anton, Jesus Paula Carvalho. Preparation of tables: Rodrigo Pinto Fernandes, Rossana Veronica Mendonza Lopez, José Antônio Orellana Turri. Initial draft of manuscript: Rodrigo Pinto Fernandes. Manuscript writing, review and approval: all authors.

## Conflicts of Interest

The authors declare no conflicts of interest.

## Synopsis

This ambispective study examined surgical approaches for endometrial carcinoma (EC) limited to the uterus (FIGO 2018 stages IA/IB) at a single institution between 2015 and 2024. Two surgical techniques were compared: systematic lymphadenectomy (LND) and sentinel lymph node (SLN) mapping. For SLN mapping, indocyanine green (ICG) dye was injected into the cervix to trace lymphatic pathways. Patients’ baseline data, surgical outcomes, and postoperative complications were collected. Propensity score matching was used to mitigate bias, comparing 84 patients in each group.

## Data Availability

The data that support the findings of this study are available on request from the corresponding author. The data are not publicly available due to privacy or ethical restrictions.
